# Differential Influence of the COVID-19 Pandemic on Mechanical Thrombectomy and Bridging Therapy for Acute Ischemic Stroke

**DOI:** 10.3389/fneur.2022.852423

**Published:** 2022-03-22

**Authors:** Dandan Geng, Xueqian Xu, Xiaoqian Luan, Linan Qiu, Liuzhu Chen, Jiahao Chen, Beilan Wu, Minjie Xu, Akmal Ergashev, Wenjie Tang, Jia Li

**Affiliations:** ^1^Department of Neurology, The First Affiliated Hospital of Wenzhou Medical University, Wenzhou, China; ^2^Wenzhou Medical University School of Mental Health, Wenzhou, China; ^3^Department of General Surgery, The First Affiliated Hospital of Wenzhou Medical University, Wenzhou, China; ^4^The First School of Clinical Medicine, Wenzhou Medical University, Wenzhou, China

**Keywords:** COVID-19, acute ischemic stroke, mechanical thrombectomy, bridging thrombolysis, functional prognosis

## Abstract

**Background:**

The coronavirus disease 2019 (COVID-19) pandemic is having a dramatic impact on acute stroke care. Its effects may accompany stroke care for a long time. We compared the treatment, short-term and long-term functional outcomes of patients with AIS from 2019 to 2020. Our objective was to evaluate the effect of COVID-19 epidemic on mechanical thrombectomy (MT) in patients in our hospital.

**Methods:**

We collected information on subjects treated with MT in 2019–2020, including age, sex, time from the onset to arterial sheath insertion, time from the onset to recanalization, the rate of lung infection and hemorrhagic transformation, modified Rankin scale (mRS), NHISS, and ASPECTS.

**Results:**

The number of patients with MT decreased significantly by 26.6% in 2020 (*p* = 0.025). The pretreatment ASPECTS score for 2020 was significantly higher than 2019 (*p* = 0.004). Besides, the patients were more likely to develop lung infection (65 vs. 54.1%, *p* = 0.042) and had a higher risk of hemorrhagic transformation (47.4% vs. 30.4%, *p* = 0.005) in 2019. The discharged mRS reflected the worse short-term functional prognosis of patients with MT in 2019 (66 vs. 44.9%, *p* = 0.046). In the subgroup analysis of bridging thrombolysis (BT), more patients with BT are expected to have a poor short-term functional prognosis in 2020, according to the discharged mRS (62.5 vs. 37.5%, *p* = 0.024). However, there was no difference in mRS at 180 days between the two groups (*p* = 0.094).

**Conclusion:**

For patients with MT, both short- and long-term functional outcomes were not significantly affected due to the mild condition of patients admitted to hospital in 2020. For patients with BT, the COVID-19 pandemic has prolonged the green channel time of stroke, leading to a poor short-term functional prognosis of patients with stroke in the pandemic period. There was no difference in the effectiveness of direct MT and BT during the COVID-19 pandemic.

## Introduction

Stroke, a cerebrovascular accident, prevails in all patient populations and can be a significant cause of morbidity and mortality. Every year, an estimated two million people suffer from a stroke (1). Numerous treatments for ischemic strokes are available, primarily pharmacological and mechanical therapies. Mechanical thrombectomy (MT) has gradually become a widely used therapy for stroke treatment. It is reported that MT substantially extended the therapeutic window up to 24 h in selected patients (2). Coronavirus disease 2019 (COVID-19) is a pandemic that can cause systemic complications and have a high mortality rate, which has defied health care systems worldwide (3). The epidemic has had a severe impact on the prevalence and treatment of stroke. A study reported a decrease of 21% in MT cases in the COVID-19 pandemic in France (4). Other research has shown a 21% decrease in MT interventions performed by the outpatient referral hospital in the United Kingdom (5). In some research, COVID-19 has not affected the short-term outcomes of patients with MT. However, some studies have reported that COVID-19 impacted the functional development of acute ischemic stroke (AIS) care compared to control periods in 2019 (6–13). The influence of the COVID-19 pandemic on stroke and MT is still debated. Compared with direct MT, there is still no clear risk or a benefit profile of intravenous thrombolysis prior to MT (bridging thrombolysis) during the outbreak. Besides, there was no long-term follow-up [such as 180 days of the modified Rankin scale (mRS)] to estimate the long-term curative effect of MT during the epidemic. After 180 days of discharge, patients with stroke will enter the platform stage of rehabilitation, and their physical recovery level will be relatively constant. Therefore, evaluating 180-day follow-up results can make a more tailored treatment plan for clinical practice.

We have watched the treatment and prognosis of patients with MT treatment during the outbreak. The situation of the patients treated with MT between 2019 and 2020 was compared. In addition, we sought to investigate and compare the number and treatment rates of hospitalized patients treated with direct MT and bridging thrombolysis (BT), including short- and long-term outcomes during the COVID-19 pandemic in 2020. We hope this will contribute to neurologists choosing the best treatment to help patients during the outbreak of COVID-19 in the future.

## Methods

### Study Population

This was a single-center, observational, retrospective study conducted at the largest cerebrovascular disease medical center in the Wenzhou region (the First Hospital of Wenzhou Medical University with an independent neuro-emergency clinic and a mature green ISA channel (with complete information construction of stroke and rapid diagnostic, imaging, and laboratory testing time). Data from January 2019 to December 2020 were examined, covering patients admitted to the hospital 24 h after the symptom onset and treated with reperfusion. The patients included in the data analysis met the following criteria: (1) the patients above the age of 18; (2) clinical diagnosis of AIS confirmed by CTA, MRA, or DSA, with symptoms lasting more than 30 min and no improvement before treatment; (3) all the patients were treated with MT. We excluded all the patients due to the following exclusion criteria: 388 patients were treated with IVT alone or pharmacological endovascular treatment (EVT), 80 patients were not followed up, or lacked critical outcome data at 180 days. Finally, 326 people were enrolled in the main analysis. The study was approved by the Ethics Committee of the First Affiliated Hospital of Wenzhou Medical University (YS2018002) and conducted in accordance with the ethical guidelines of the declaration of Helsinki in 1975. All the participants or their guardians were well aware of our study and signed written informed consent.

### Clinical Data Collection

We collected information on subjects treated with MT in 2019–2020. The steps of information collection were as follows: First, we obtained data from the green channel of stroke, including age, sex, time from the onset to arterial sheath insertion, time from the onset to recanalization, the rate of lung infection and hemorrhagic transformation, etc. The data of modified Rankin scale (mRS), pretreatment NHISS, and discharged NHISS were collected from inpatient medical records of the neurology department. Short-term prognosis-dependent mRS assessment at discharge was divided into two components, 3–6 scores for functional dependence or death at discharge and 0–2 scores for suggesting a better prognosis. Long-term follow-up depends on mRS assessment at 180 days.

During the Spring Festival, more than 90,000 people from Wuhan returned to Wenzhou, making the city hard hit by the coronavirus (14). Wenzhou issued an official decree, beginning to restrict social activities on February 2, 2020. The strictest restrictions were canceled in March, but other limits continued throughout the year. So, the residents in the area experienced huge changes in their lives, bringing considerable changes to the healthcare system from 2019 to 2020.

### Statistical Analysis

Categorical variables were expressed in proportion, and the chi-square test was used for differences between groups. According to the distribution of continuous data, mean ± SD was used to describe the normal distribution, and Student's T-test was used to evaluate the difference between groups. Median (interquartile spacing, IQR) and Mann–Whitney U tests were used for skewness distribution. We created an Excel line chart to highlight the difference in time from the onset to arterial sheath insertion between 2019 and 2020. The correlation analysis graph in Graphpad was used to compare the correlation of short- and long-term functional outcomes with the time from the onset to arterial sheath insertion in the pandemic. A two-tailed *p* < 0.05 was considered statistically significant and was calculated using IBM SPSS statistical software Version 26 for Windows.

## Result

### Baseline Characteristics of Patients With MT

**Figure 1:**
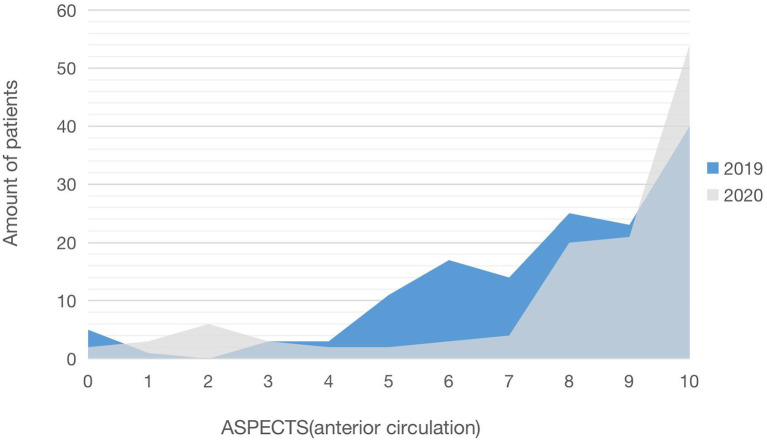
The difference of an ASPECTS score (2019) before and (2020) after COVID-19.

As shown in Table 1, we compared and analyzed the differences in the baseline between the patients treated with MT before and after COVID-19. The total number of the patients with MT in 2020 was 138, a considerable decrease from 188 in 2019. Since the outbreak of COVID-19 epidemic and the implementation of restrictions in Wenzhou, the number of patients with MT has decreased significantly by 26.6%, and the average number of monthly patients with MT has decreased compared to 2019 (11.5 ± 3.9 vs. 15.7 ± 4.6, *p* = 0.025). Age (*p* = 0.276), gender (*p* = 0.516), pretreatment NHISS (*p* = 0.494), the number of patients receiving BT treatment (*p* = 0.185), the time from the onset to arterial sheath insertion (*p* = 0.091), and the time from the onset to recanalization (*p* = 0.082) did not show any significant differences. When we compared the ASPECTS scores of the anterior circulation between the years 2019 and 2020, we found a significant difference [median (IQR): 8 (6–10) vs. 9 (8–10), *p* = 0.004]. In 2020, there were substantially more patients with MT with ASPECTS 8-10 scores than in 2019 (68.8 vs. 46.8%) (Figure 1).

**Table 1:** Baseline characteristics and outcome evaluation of patients with MT (2019) before and (2020) after COVID-19.

**Parameters**	**2019** **(*n* = 188)**	**2020** **(*n* = 138)**	***p*** **value**
MT patients ( monthly average) (mean ± SD)	15.7 ± 4.6	11.5 ± 3.9	0.025^*^
Age (mean ± SD)	66.0 ± 12.9	67.5 ± 11.4	0.276
Male, *n* (%)	130 (69.1%)	100 (70.6%)	0.516
Time from onset to arterial sheath insertion (h), median (IQR)	5.6 (4.2–7.5)	5.9 (4.6–8.1)	0.091
Time from onset to recanalization (h), median (IQR)	6.5 (5.1–8.2)	7.0 (5.5–9.0)	0.082
Lung infection, *n* (%)	117 (65.7%)	66 (54.1%)	0.042^*^
Hemorrhagic transformation, *n* (%)	74 (47.4%)	34 (30.4%)	0.005^*^
Bridging thrombolysis, *n* (%)	56 (29.8%)	32 (23.2%)	0.185
mTICI 2b/3, *n* (%)	123 (85.4%)	105 (89.1%)	0.034^*^
Occlusion after recanalization, *n* (%)	17 (12.1%)	10 (9.0%)	0.426
**TOAST mechanism**			0.005^*^
LAA, *n* (%)	88 (51.8%)	75 (60.5%)	
CE, *n* (%)	71 (41.8%)	31 (25.0%)	
SVO, *n* (%)	1 (0.6%)	4 (3.2%)	
Others, *n* (%)	10 (5.9%)	14 (11.3%)	
Anterior circulation, *n* (%)	128 (77.1%)	93 (79.5%)	0.879
ASPECTS (anterior circulation only), median (IQR)	8.0 (6.0–10.0)	9.0 (8.0–10.0)	0.004^*^
Pretreatment NHISS (mean ± SD)	13.3 ± 6.5	14.1 ± 7.9	0.494
NHISS (discharged), median (IQR)	5.5 (2.0–12.0)	4.5 (1.0–10.0)	0.502
**mRS (discharged)**, ***n*** **(%)**			0.046^*^
0–2	64 (34.0%)	76 (55.1%)	
3–6	124 (66.0%)	62 (44.9%)	
**mRS (180 days)**			0.464
0–2	98 (71.0%)	72 (66.7%)	
3–6	40 (29.0%)	36 (33.3%)	

*MT, mechanical thrombectomy; mTICI, modified treatment in cerebral infarction; TOAST, trial of ORG 10172 in acute stroke; NIHSS, The National Institutes of Health Stroke Scale; mRS , modifed Rankin scale*.

* p values considered statistically significant; ^*^p < 0.05.

### Postoperative Status and Outcome Evaluation

We further discovered a substantial difference in a prognosis between patients with MT before and after COVID-19. Specifically, there were statistical differences in postoperative lung infection, hemorrhage transformation, successful reperfusion rate (mTICI 2b/3) within 2 years. According to the findings, the patients with MT in 2019 were more likely to develop lung infection (65 vs. 54.1%, *p* = 0.042) and had a higher risk of hemorrhagic transformation (47.4 vs. 30.4%, *p* = 0.005). The rate of successful reperfusion (mTICI 2b/3) in 2019 was substantially lower than that in 2020 (85.4 vs. 89.1%, *p* = 0.034). We examined the discharged mRS evaluation and followed mRS assessment for 180 days to determine the short- and long-term outcomes of patients with MT. We found the discharged mRS reflected the patients with MT in 2019 had a worse short-term functional prognosis (66.0 vs. 44.9%, *p* = 0.046). But no significant difference exists in long-term functional outcomes between 2019 and 2020 (*p* = 0.464) (Table 1).

### Subgroup Analysis of Bridging Thrombolysis

**Figure 2:**
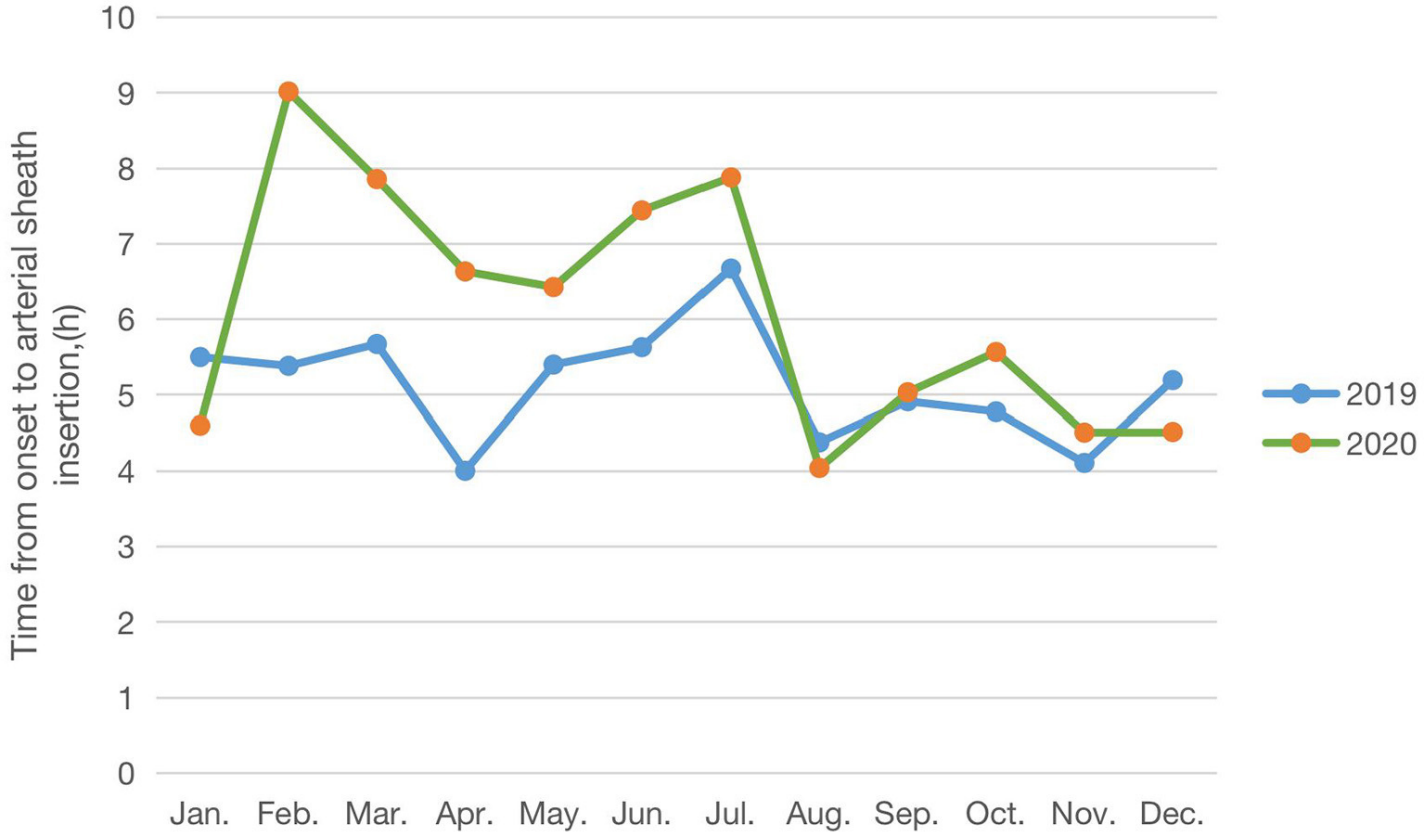
Time from the onset to an arterial sheath insertion trend of patients with BT (2019) before and (2020) after COVID-19.

**Figure 3:**
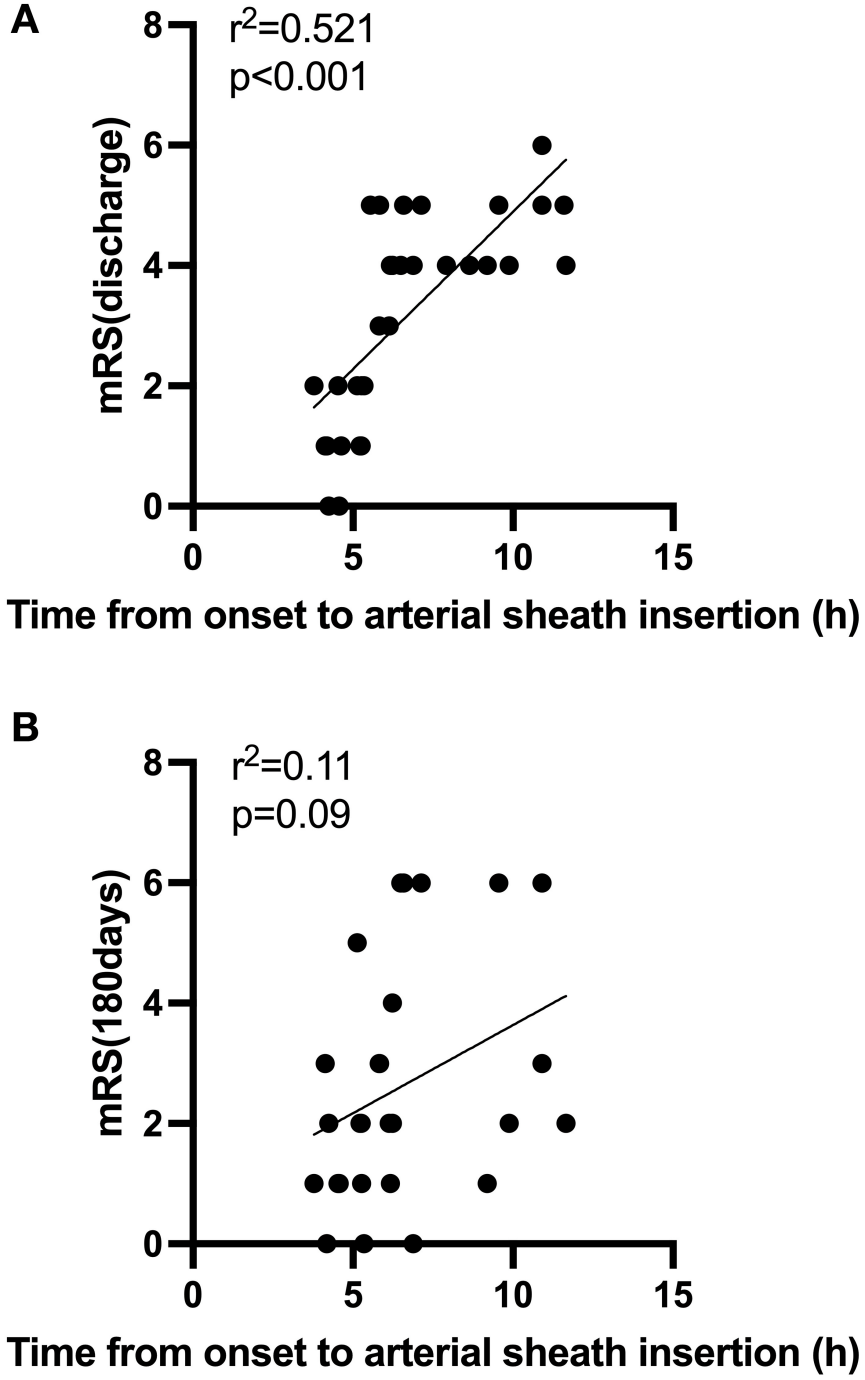
**(A,B)** Correlation between time from the onset to arterial sheath insertion and functional outcomes during the COVID-19 period.

In Table 2, a comparative analysis of patients with BT before and after COVID-19 is performed. We observed an increase of 20.6% in male patients in 2020 compared to 2019 (84.4 vs. 60.7%, *p* = 0.016). In terms of time, from the onset to arterial sheath insertion between 2019 and 2020, we observed an increase from a median (IQR) of 4.9 (4.0–5.9) h to 5.8 (4.9–6.2) h (*p* = 0.012). The time from the onset to arterial sheath insertion in each month showed that February, March, April, June, July 2020 were significantly higher than 2019 (Figure 2). That trend disappeared in August. In addition, the time from the onset to recanalization was longer in general in 2020 than in 2019 [median (IQR): 6.3 (5.1–7.0) h vs. 5.8 (4.7–7.1) h, *p* = 0.044]. There were also no significant differences in pretreatment NHISS (*p* = 0.202), discharged NHISS (*p* = 0.823), postoperative lung infection (*p* = 0.06), hemorrhage transformation (*p* = 0.444), and successful reperfusion rate (mTICI 2b/3) (*p* = 0.605) of patients with BT in 2019 and 2020. However, more patients with BT are expected to have a poor short-term functional prognosis in 2020, according to the discharged mRS (62.5 vs. 37.5%, *p* = 0.024). Through follow-up surveys, we found mRS at 180 days also showed a trend toward poorer long-term functional outcomes in more patients with BT in 2020, although there was no statistical difference (40.7 vs. 22.2%, *p* = 0.094). In addition, an interesting finding was that the longer the time from the onset to arterial sheath insertion, the worse the short-term functional prognosis of patients with BT, showing a positive correlation (*R*^2^ = 0.521, *p* < 0.001; Figure 3A), but there was a lack of correlation between a long-term functional prognosis and the time from the onset to arterial sheath insertion (*p* = 0.09; Figure 3B).

**Table 2:** Subgroup analyses for patients with BT treatment (2019) before and (2020) after COVID-19.

**Parameters**	**2019** **(*n* = 56)**	**2020** **(*n* = 32)**	***p*** **value**
Age (mean ± SD)	64.4 ± 12.1	64.4 ± 10.0	0.999
Male, *n* (%)	34 (60.7%)	27 (84.4%)	0.016^*^
Time from onset to arterial sheath insertion (h), median (IQR)	4.9 (4.0–5.9)	5.8 (4.9–6.2)	0.012^*^
Time from onset to recanalization (h), median (IQR)	5.8 (4.7–7.1)	6.3 (5.1–7.0)	0.044^*^
Lung infection, *n* (%)	33 (61.1%)	11 (39.3%)	0.060
Hemorrhagic transformation, *n* (%)	20 (40.5%)	9 (36.0%)	0.444
mTICI 2b/3, *n* (%)	36 (92.3%)	22 (95.7%)	0.605
Occlusion after recanalization, *n* (%)	2 (5.1%)	2 (9.1%)	0.615
**TOAST mechanism**			0.047^*^
LAA, *n* (%)	27 (54.0%)	16 (55.2%)	
CE, *n* (%)	22 (44.0%)	8 (27.6%)	
SVO, *n* (%)	0 (0.0%)	1 (3.4%)	
Others, *n* (%)	1 (2.0%)	4 (13.8%)	
Anterior circulation, *n* (%)	35 (76.1%)	22 (84.6%)	0.677
ASPECTS (anterior circulation only), median (IQR)	9.0 (6.0–10.0)	9.0 (8.0–10.0)	0.519
Pretreatment NHISS (mean ± SD)	12.7 ± 6.2	10.5 ± 7.5	0.202
NHISS (discharged), median (IQR)	3.0 (1.0–6.8)	2.0 (1.0–12.3)	0.823
**mRS (discharged)**, ***n*** **(%)**			0.024^*^
0–2	35 (62.5%)	12 (37.5%)	
3–6	21 (37.5%)	20 (62.5%)	
**mRS (180 days)**, ***n*** **(%)**			0.094
0–2	35 (77.8%)	16 (59.3%)	
3–6	10 (22.2%)	11 (40.7%)	

*mTICI, modified treatment in cerebral infarction; TOAST, trial of ORG 10172 in acute stroke; NIHSS, The National Institutes of Health Stroke Scale; mRS, modified Rankin scale*.

* p values considered statistically significant; ^*^p < 0.05.

### Direct Mechanical Thrombectomy vs. Bridging Thrombolysis

In Table 3, we did a comparison between direct MT and BT during the COVID-19 epidemic. According to the findings, the time from the onset to arterial sheath insertion [median (IQR): direct MT: 6.6 (4.8–8.6) h; BT: 5.3 (4.3–6.2) h; *p* = 0.023], pretreatment NHISS (direct MT: 15.6 ± 7.6; BT: 10.5 ± 7.5; *p* = 0.009) showed some significant differences between direct MT and BT. Lung infection, hemorrhagic transformation, successful reperfussion (mTICI 2b/3) rates after treatment, and the discharged NHISS did not differ between both groups, neither a good clinical outcome at discharge (direct MT: 47.2%; BT: 37.5%, *p* = 0.418) and 180 days (direct MT: 69.1%; BT: 59.3%, *p* = 0.346), nor for mortality (direct MT: 19.8%; BT:15.6%, *p* = 0.611).

**Table 3:** Comparison of bridging thrombolysis with direct MT during the COVID-19 epidemic.

**Parameters**	**Direct mechanical** **thrombectomy** **(*n* = 106)**	**Bridging** **thrombolysis** **(*n* = 32)**	***p*** **value**
Age (mean ± SD)	68.5 ± 11.7	64.4 ± 10.0	0.079
Male, *n* (%)	73 (68.9%)	27 (84.4%)	0.085
Time from onset to arterial sheath insertion (h), median (IQR)	6.6 (4.8–8.6)	5.3 (4.3–6.2)	0.023^*^
Time from onset to recanalization (h), median (IQR)	8.0 ± 3.5	7.0 ± 3.5	0.19
Lung infection, *n* (%)	55 (58.5%)	11 (39.3%)	0.073
Hemorrhagic transformation, *n* (%)	25 (28.7%)	9 (36.0%)	0.486
mTICI 2b/3, *n* (%)	83 (93.3%)	22 (95.7%)	0.660
Occlusion after recanalization, *n* (%)	8 (9.0%)	2 (9.1%)	0.988
Pretreatment NHISS (mean ± SD)	15.6 ± 7.6	10.5 ± 7.5	0.009^*^
NHISS (discharged), median (IQR)	5.0 (1.0–10.0)	2.0 (1.0–12.3)	0.509
**mRS (discharged)**, ***n*** **(%)**			0.418
0–2	50 (47.2%)	12 (37.5%)	
3–6	56 (52.8%)	20 (62.5%)	
**mRS (180 days)**, ***n*** **(%)**			0.346
0–2	56 (69.1%)	16 (59.3%)	
3–6	25 (30.9%)	11 (40.7%)	
Mortality (180 days), *n* (%)	16 (19.8%)	5 (15.6%)	0.611

*mTICI, modified treatment in cerebral infarction; TOAST, trial of ORG 10172 in acute stroke; NIHSS, The National Institutes of Health Stroke Scale; mRS, modified Rankin scale*.

* p values considered statistically significant; ^*^ p < 0.05.

## Discussion

Acute stroke treatment requires effectiveness, as early as possible to dredge blood vessels, can save more brain tissue and brain cells, and achieve a better prognosis, so time is the brain. At present, the main treatment methods are intravenous thrombolysis (IVT), direct mechanical thrombectomy (MT), and bridging thrombolysis (BT). This article provides accurate data on patients treated with MT throughout the year during the COVID-19 pandemic in 2020, compared to the same data in 2019.

Firstly, we found that the number of patients with acute stroke treated with MT decreased significantly in 2020, consistent with the decline in the number of patients seen in medical systems worldwide during the COVID-19 pandemic. A similar research in the UK showed the number of patients admitted to hospital for stroke fell by 27.7% in MT cases in the COVID-19 pandemic (15). Another research in America reported a significant reduction of the MT procedure in the COVID-19 pandemic (16). The most likely reason is that, due to the restriction policy under the epidemic situation, the time for patients to arrive at the medical center increases, so the number of patients exceeding the treatment time window increases. In addition, the stroke nursing center implements the strict screening of MT for eligible patients. Finally, the number of patients who meet the strict guidelines of MT decreases (4, 5). Other possible reasons are the increase in healthy lifestyle due to the COVID-19 epidemic restriction policy, which keeps people away from hospital (17, 18), or residents in areas around Wenzhou, where the restrictions are in place, may opt for less busy local medical facilities nearby, are not stroke treatment facilities. Doctors in these services may diagnose fewer strokes or not refer all cases to our hospital. Besides, some patients with mild stroke symptoms are unwilling to arrive at the hospital or emergency room to avoid exposure to COVID-19 (15, 16). Therefore, we need to explain and publicize to the public, which is not a problem because the hospital has taken standard preventive measures to ensure that patients and medical personnel are protected. In addition, it is necessary to establish a more reasonable stroke care network, allocate resources reasonably, and reduce the impact of the COVID-19 pandemic on the medical system.

Secondly, in our study, the patients admitted in 2019 had a worse ASPECTS score of anterior circulation, more postoperative lung infections, higher hemorrhagic transformation, and a lower successful regenerative rate than the patients admitted in 2020. Following this, the short-term functional outcomes were worse in 2019, but there was no difference in long-term functional outcomes at 180 days between the 2 years. The main reason is that the patients accepted in 2019 had a relatively large infarct area, severe condition, and more postoperative complications, so the short-term functional prognosis was poor. Some studies have proved that core volume, infection, hemorrhagic transformation, and reperfusion rate were significant predictors of good outcomes (19–22). Patients treated with MT had smaller infarcts during the pandemic, so they had a higher chance of benefiting from MT.

Thirdly, the subgroup analyses for patients with BT treatment showed different results. We found that the time from the onset to arterial sheath insertion increased in 2020, meaning that patients had a longer time from the onset to arrive at hospital or a longer hospital stay before endovascular treatment. More importantly, the time from the onset to arterial sheath insertion was observed to increase in February, March, April, June, and July 2020. This trend is consistent with our previous study on IVT during epidemics (23). The main reason may be the outbreak in recent months, Wenzhou's implementation of strict restrictions, affects and delays the green channel stroke. To maintain social distancing for everyone following hospital pandemic preparedness protocols, and take isolation measures, rapid assessment, screening, and treating patients for stroke, all of which can systematically reduce the rapidity that patients receive treatment (24). Another reason is the shortage of doctors in stroke centers due to the pandemic's overstretched health care resources. Balancing the epidemic control and the efficiency of the green channel is an urgent problem for hospitals.

In addition, data showed that the short-term functional prognosis of patients with BT during the pandemic was worse than in 2019, and there was no difference in a long-term functional prognosis. Considering that there was no difference in the preoperative infarct core area and postoperative complications between the 2 years, this may have amplified the effect of the time from the onset to arterial sheath insertion on short-term functional outcomes, resulting in worse treatment outcomes in 2020. An interesting finding was that the longer the time from the onset to arterial sheath insertion, the worse the functional outcome of discharge. However, it was not associated with long-term functional outcomes. The possible reason is that “time is brain” is reflected in the process of BT treatment. The most famous New England Journal published the results of MR CLEAN's study in 2015, and the analysis showed that the time when patients started endovascular therapy was highly correlated with the prognosis (25). With the delay of treatment time, the likelihood of a patient's good prognosis plummets. Therefore, a rapid response system and a 40-min strategy (diagnosis, imaging, and laboratory testing time < 40 min) should be established to shorten the hospital preparation time according to the stroke treatment map provided by China Stroke Prevention Project Committee (CSPPC) (26).

Finally, in the context of the COVID-19 pandemic in 2020, this study concluded with a comparison of direct patients with MT and patients with BT. There was no difference in lung infection, recanalization rates, bleeding complications, functional recovery, and mortality. This is consistent with the research of Sebastian Bellwald, MD* et al. (27). A pre-pandemic meta-analysis of five randomized controlled trials also showed no difference in the magnitude of treatment effect between patients who received MT directly and those who received IVT and MT simultaneously (28). So there was no additional benefit from IVT prior to MT even during an epidemic. From the perspective of clinical practice, for patients whose treatment was delayed due to COVID-19, endovascular treatment should be as early as possible. If endovascular treatment is temporarily unavailable, patients may have access to at least one method of recanalization therapy ahead of time. Therefore, medical institutions at all levels should combine the actual conditions of patients and hospitals to select treatment strategies conducive to improving patients' clinical outcomes and reducing the pandemic's impact on stroke.

## Conclusion

In summary, our study supported the number of patients with AIS treated with MT decreased significantly in 2020. The COVID-19 pandemic especially prolonged the time of green channel for stroke, which led to the worse short-term prognosis of patients with BT. In addition, there was no difference between direct MT and BT in a clinical prognosis during the pandemic. Therefore, building rapid green channels, ensuring adequate medical resources, improving care systems, and choosing the most appropriate treatment strategy will effectively improve patient outcomes during the pandemic.

## Data Availability Statement

The raw data supporting the conclusions of this article will be made available by the authors, without undue reservation.

## Ethics Statement

This study was reviewed and approved by the Ethics Committee of the First Affiliated Hospital of Wenzhou Medical University (YS2018002). Written informed consent was obtained from all participants, or the participants' legal guardian/next of kin, for participation in this study. Written informed consent was obtained from the individual(s) for the publication of any potentially identifiable images or data included in this article.

## Author Contributions

JL and WT designed the study and wrote the protocol. DG, XX, XL, LQ, LC, JC, BW, MX, and AE conducted literature searches and provided summaries of previous research studies. XL conducted the statistical analysis. DG wrote the first draft of the manuscript and all the authors contributed to and have approved the final manuscript.

## Funding

This research was supported by a Grant from Wenzhou Municipal Sci-Tech Bureau Program (Y2020421).

## Conflict of Interest

The authors declare that the research was conducted in the absence of any commercial or financial relationships that could be construed as a potential conflict of interest.

## Publisher's Note

All claims expressed in this article are solely those of the authors and do not necessarily represent those of their affiliated organizations, or those of the publisher, the editors and the reviewers. Any product that may be evaluated in this article, or claim that may be made by its manufacturer, is not guaranteed or endorsed by the publisher.
